# Gene function in early mouse embryonic stem cell differentiation

**DOI:** 10.1186/1471-2164-8-85

**Published:** 2007-03-29

**Authors:** Kagnew Hailesellasse Sene, Christopher J Porter, Gareth Palidwor, Carolina Perez-Iratxeta, Enrique M Muro, Pearl A Campbell, Michael A Rudnicki, Miguel A Andrade-Navarro

**Affiliations:** 1 Ontario Genomics Innovation Centre, Ottawa Health Research Institute, 501 Smyth, Ottawa, ON, K1H 8L6, Canada

## Abstract

**Background:**

Little is known about the genes that drive embryonic stem cell differentiation. However, such knowledge is necessary if we are to exploit the therapeutic potential of stem cells. To uncover the genetic determinants of mouse embryonic stem cell (mESC) differentiation, we have generated and analyzed 11-point time-series of DNA microarray data for three biologically equivalent but genetically distinct mESC lines (R1, J1, and V6.5) undergoing undirected differentiation into embryoid bodies (EBs) over a period of two weeks.

**Results:**

We identified the initial 12 hour period as reflecting the early stages of mESC differentiation and studied probe sets showing consistent changes of gene expression in that period. Gene function analysis indicated significant up-regulation of genes related to regulation of transcription and mRNA splicing, and down-regulation of genes related to intracellular signaling. Phylogenetic analysis indicated that the genes showing the largest expression changes were more likely to have originated in metazoans. The probe sets with the most consistent gene changes in the three cell lines represented 24 down-regulated and 12 up-regulated genes, all with closely related human homologues. Whereas some of these genes are known to be involved in embryonic developmental processes (e.g. *Klf4, Otx2, Smn1, Socs3, Tagln, Tdgf1*), our analysis points to others (such as transcription factor *Phf21a*, extracellular matrix related *Lama1 *and *Cyr61*, or endoplasmic reticulum related *Sc4mol *and *Scd2*) that have not been previously related to mESC function. The majority of identified functions were related to transcriptional regulation, intracellular signaling, and cytoskeleton. Genes involved in other cellular functions important in ESC differentiation such as chromatin remodeling and transmembrane receptors were not observed in this set.

**Conclusion:**

Our analysis profiles for the first time gene expression at a very early stage of mESC differentiation, and identifies a functional and phylogenetic signature for the genes involved. The data generated constitute a valuable resource for further studies. All DNA microarray data used in this study are available in the StemBase database of stem cell gene expression data [1] and in the NCBI's GEO database.

## 1. Background

There is a growing interest in the identification of genes responsible for stem cell phenotypes and behaviour, due largely to the envisioned potential of the field of stem cell therapeutics. However, relatively few genes have been identified as associated with stem cell generation or maintenance [2]. Such genes are likely to be important in deriving methods to control and direct the differentiation of stem cells for therapy, for example by expanding a stem cell culture or by directing differentiation towards a targeted cell type. The knowledge of the genes and processes involved in stem cell control is particularly sparse for embryonic stem cells (ESCs), though ESCs are the most interesting for therapy as they have the potential to differentiate into all cell types.

DNA microarrays allow the simultaneous profile of thousands of mRNA transcripts, thereby providing a general overview of the state of gene expression in a cell sample. Here, we have used DNA microarrays to identify genes, and thus cellular processes, that might be important to the differentiation of mouse ESCs (mESCs). We have profiled samples from three biologically equivalent but genetically distinct mESC lines (J1, R1, and V6.5) undergoing *in vitro *differentiation into embryoid bodies (EBs). Upon removal of LIF (leukemia inhibitory factor), and in the absence of murine embryonic feeder cells, mESCs can be grown as unattached spheres termed EBs, which contain mesodermal, ectodermal, and endodermal cells [3]. EB formation from mESCs has been proposed as a model for early embryonic development in terms of differentiation capacity, morphological changes, and inductive signaling events that drive these changes [4,5].

Measurements of gene expression were taken from biological triplicates during differentiation at 11 time points spanning a period of two weeks following LIF removal. Measures were taken every six hours for the first 24 hours, then at increasing intervals, with time points at: 0 h (undifferentiated mESCs), 6 h, 12 h, 18 h, 24 h, 36 h, 48 h, 4 d, 7 d, 9 d, and 14 d. The cells' mRNA content was assayed at each time point using the Affymetrix MOE430A/B GeneChip set. Our hypothesis is that genes important for the control of the differentiation process should show large expression changes during the initial period of differentiation.

We found that during the initial 12 hour period the gene expression of transcription factors is still relatively unchanged respect to the undifferentiated mESCs in all three time courses. We conclude that this period represents cells not yet differentiated, and is, therefore, appropriate for the study of the early changes in gene expression leading to differentiation.

To confirm that the gene expression data of the first 12 hours of the time series pinpoints to genes related to mESC differentiation, we performed a heuristic method for the analysis of the DNA microarray data that yielded the genes with the largest changes in expression in that period in two or three of the cell lines analyzed.

We classified the changes in gene expression in that period with respect to gene function and phylogenetic profile of the corresponding protein products. Our results showed a significant abundance of proteins related to transcription regulation, intracellular signaling, and the cytoskeleton; and enrichment in protein sequences whose phylogenetic distribution of homologs indicated that their evolutionary origin occurred along the metazoan lineage.

Finally, we applied a more standard method of DNA microarray data analysis (Significance Analysis of Microarrays; SAM), to select 36 genes with the most consistent expression changes during the initial 12 hours of the time series in two or three of the cell lines analyzed. We discuss the functions and possible relations of those 36 genes with mESC differentiation, which includes genes known to be involved in embryonic differentiation processes but also several never before linked to those processes.

Our study differs from previous genomic scale gene expression surveys of human and mouse ESC differentiation [6,7] in that it examines in parallel the differentiation of three cell lines using a greater number of time points, starting at a much earlier point after the initiation of differentiation. We therefore expect that our findings expand upon the information generated in those studies.

## 2. Results

### 2.1. Demarcation of an early period of differentiation

In order to identify early pre-differentiation changes in mESC gene expression, we defined the period for each of the three time series in which cell populations were still predominantly undifferentiated ESCs. We reasoned that the genes involved in triggering differentiation would be among those whose expression changed the most during that period. We performed two analyses to define this early period.

**Figure 1:**
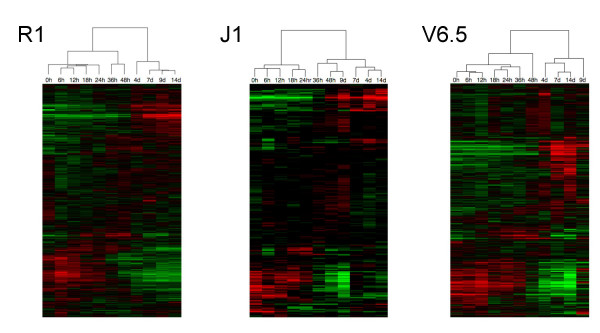
**Clustered heat maps for differentiation time-course experiments in three mouse ES cell lines: R1, J1, and V6.5**. Expression values for 1,605 probe sets, corresponding to 1,327 transcription factor genes (according to protein domain analysis), were extracted from data from the MOE430A microarray for 11 time points from 0 hours to 14 days. The data for each cell line were mean-centered by probe set and by array, and clustered using Cluster 3.0 (University of Tokyo). Clusters were visualized using Java TreeView [72].

Firstly, we performed two-dimensional hierarchical clustering of the signal values of 1,605 probe sets corresponding to 1,327 known or predicted transcription factors against the eleven time points for each cell line (Figure 1). The reasoning behind this was to identify for each cell line the period during which changes in the genetic regulatory network were yet relatively small. We identified the time points that clustered closely with the first (0 h) time points, when the culture consists primarily of ESCs, and classified these as the early period. In the J1 and V6.5 lines, the expression patterns at 0, 6 and 12 hours clustered together; in R1, the 0 h pattern formed a cluster with the 6, 12, 18, and 24 hour patterns.

**Figure 2:**
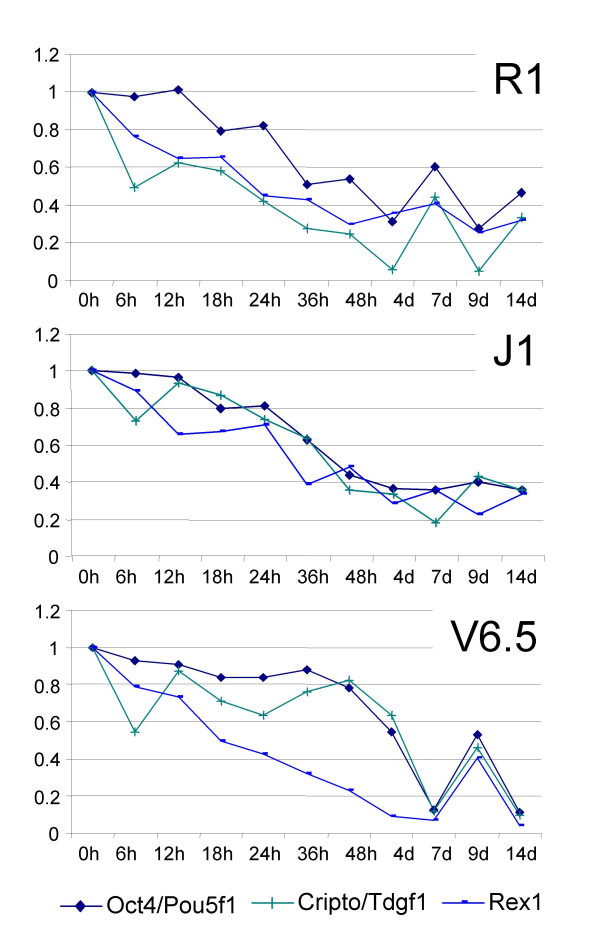
**Marker genes (*Oct4/Pou5f1*, *Cripto/Tdgf1*, and *Rex1*) expression pattern in the three time series**. The identifiers of the probe sets used were 1417945_at (for *Oct4/Pou5f1*), 1450989_at (for *Cripto/Tdgf1*), and 1418362_at (for *Rex1*). Signal values were normalized to the value at 0 h.

Secondly, in order to define the parts of the three time series corresponding mainly to an ESC population, we examined the signal values of probe sets corresponding to known markers of ESCs. Out of ten marker genes examined, only three (*Oct4/Pou5f1 *[8], *Cripto/Tdgf1 *[9], and *Rex1 *[10]) had high initial values of expression which decreased gradually over time in all three cell lines. We observed that the expression of these three marker genes stayed between 50%-100% of their 0 h value at 6 h and 12 h (Figure 2), suggesting that the culture consists primarily of ESC in that time range.

These two results suggest that the first three points of each time series (representing the first 12 hours) can be used to identify genes whose expression changes significantly in the early stages of differentiation, before wide scale alterations of gene expression. This observation is consistent with, and conservative relative to, a previous study of mESC showing that a major loss of pluripotency occurs within the first 24 hours of differentiation into EBs [7].

Based on this result, we focused on probe sets showing consistent changes of expression in the first 12 hours of the time course in at least two of the three cell lines. These genes are likely to include those involved in processes controlling early differentiation.

### 2.2. Broad functional analysis

To confirm that the gene expression data of the first 12 hours of the time series can be used to detect genes related to mESC differentiation, we studied the properties of genes related to large or small expression changes in that period using a heuristic approach.

We first pooled together and normalized the data from the two GeneChips used (MOE430A and MOE430B) (see Methods). Then, we pre-selected the probe sets that were expressed in any of the 0 h, 6 h, or 12 h time points in all three cell types. For this we used the detection call (present (P), marginal (M), or absent (A)) generated by the Affymetrix MAS5 software, which indicates whether a transcript is detected, and by extension, whether a gene is expressed. Accordingly, we selected probe sets with at least one P call in any of the first three time points for all three time series. A total of 16,752 of the 45,139 probe sets on the MOE430A and MOE430B chips passed this filter [see Additional file 1].

**Figure 3:**
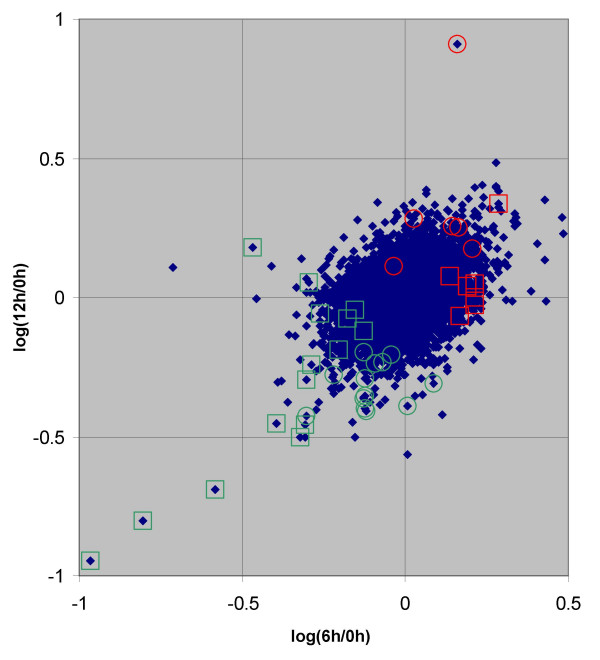
**12 h/0 h versus 6 h/0 h signal ratios for MOE430A/B probe sets**. Values are m6, the median of the three 6 h/0 h signal ratios, and m12, the median of the three 12 h/0 h signal ratios, in the three time series (section 2.2). Marked data points correspond to the 42 probe sets selected from the MOE430A data using SAM (section 2.4). Green squares: 15 probe sets down-regulated at 6 h; green circles: 13 probe sets down-regulated at 12 h; red circles: 7 probe sets up-regulated at 12 h; red squares: 7 probe sets up-regulated at 6 h. The data underlying this table are available [see Additional file 1].

Next, we computed the ratios of the signal of these probe sets at 6 h and 12 h relative to the initial time point (6 h/0 h and 12 h/0 h) in the three time series. Defining m6 and m12, as the median of the 6 h/0 h ratios and of the 12 h/0 h ratios for a given probe set in the three time series, respectively, we plotted m6 against m12 for each probe set (Figure 3). The distribution of the values indicated a small number of probe sets with particularly large fold changes.

In order to profile the functions of the genes whose expression was modified during this time period, we used the GOstat server [11], which searches for Gene Ontology (GO) terms [12] significantly under- and over-represented in a given set of probe sets or genes in relation to a reference set (see Methods for details). We first analyzed GO terms for the genes which showed the largest expression changes, regardless of the direction of the change. We sorted the probe sets by their Euclidean distance from the origin in the logs ratio graph represented in Figure 3, (0,0), which corresponds to probe sets with invariant gene expression at 0 h, 6 h, and 12 h. The top and bottom 10% of probe sets (1,675) were selected as representing the genes with the largest and smallest gene expression changes in the first 12 hours of differentiation, respectively. In the genes with the largest changes, the following GO terms were over-represented: "organogenesis" (P-value = 0.0004) and related "morphogenesis" (0.0008), "intracellular signaling cascade" (0.17), and "cell differentiation" (0.2). In contrast, for the genes with the smallest changes in expression levels, over-represented GO terms included "mitochondrion" (0.0001), "cytoplasm" (0.008), and a series of terms related to housekeeping functions, such as "ATP metabolism" (0.04) or "unfolded protein binding" (0.04).

To investigate the functions associated with a directional change in expression, we performed GOstat analysis on the 10% of probe sets with the largest (increasing expression) and smallest (decreasing expression) sums of m6 and m12 values. Over-represented GO terms in the genes with increased expression indicated "DNA binding" (0.013) and "regulation of transcription, DNA dependent" (0.09). In the genes with decreased expression, selected terms were "extracellular matrix (sensu Metazoa)" (1.63e-05), "cell communication" (0.0004), "organogenesis" (0.007), "signal transduction" (0.014), "protease inhibitor activity" (0.02), and "amino acid metabolism" (0.07).

This analysis suggests that protein production and functions related to communication between the cell and the environment were down-regulated in the time period under analysis, while the handling of genetic information was up-regulated. These observations fit with what might be expected; as differentiation is initiated, transcription factors (which bind DNA and regulate transcription) are expressed to initiate downstream changes in gene expression patterns. Meanwhile, genes for extracellular matrix components are turned off as the cells change from growing attached to the plate, to growing in floating culture; many of these same genes are related to cell communication.

### 2.3. Phylogenetic distribution

We hypothesized that genes important for stem cell function may have arisen along the metazoan lineage. Therefore, genes with large expression changes in the period studied should have a particular phylogenetic signature. To verify this, we examined the phylogenetic distribution of the homologues of the protein products assigned to each of the 16,752 probe sets selected above, and contrasted it with the gene expression changes.

**Figure 4:**
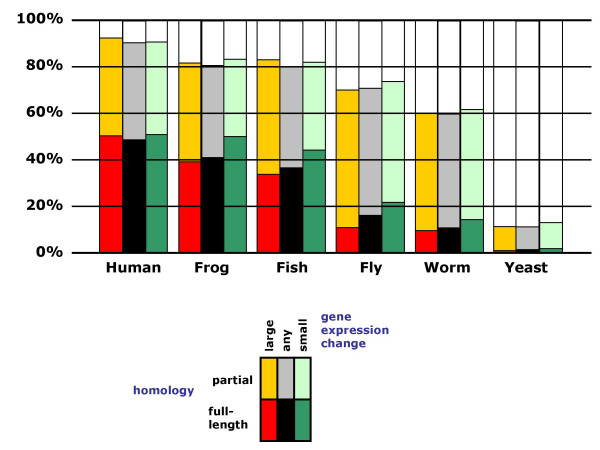
**Phylogenetic distribution of proteins associated with probe sets**. Percentage of proteins with homologues in a given organism: *Homo sapiens *(human)*, Brachydanio rerio *(fish)*, Xenopus laevis *(frog)*, Drosophila melanogaster *(fly), *Caenorhabditis elegans *(worm), and *Saccharomyces cerevisiae *(yeast). For each organism, the leftmost column indicates homologues found for proteins from the 1,675 probe sets with largest gene expression changes, the middle column those from the complete set of 16,752 probe sets, and the rightmost column those from the 1,675 probe sets with smallest gene expression changes. In each column, the dark bottom part indicates the percentage of proteins aligned along their full length (less than 30 amino acids unmatched at the N- and C-termini of both sequences), and the lighter upper part is the percentage of proteins with sequence similarity (no length restriction). Proteins were considered similar with a BLAST E-value < 1e-6. [Supplementary-material S2]

We used NetAffx [13] to identify mouse protein sequences associated with each of the probe sets, and for each mouse protein identified the most similar protein sequence in the SPtrEMBL database [14] for six model eukaryotic organisms: *Homo sapiens, Danio rerio, Xenopus laevis, Drosophila melanogaster*, *Caenorhabditis elegans*, and *Saccharomyces cerevisiae *(see Figure 4)*. C. elegans *is considered to have stem cells [15] as are the other metazoan organisms considered in this analysis.

We defined as homologues those proteins identified using BLAST with a threshold E-value of 1e-6, based on the work of Lopez-Bigas and Ouzonis [16]. Looking at data for all 16,752 probe sets (irrespective of their changes in gene expression) we saw an increase in the number of homologues between the worm and the yeast, which corresponds to the global sequence similarities between these genomes (Figure 4). Taking gene expression into account, there are no large differences in the distribution of homologues between the genes with largest and smallest gene expression changes.

However, sequence similarity between two proteins often covers only a small fraction of the total length of the sequences being compared. This is not surprising if one considers that the proteins analyzed have multiple domains some of them present in many proteins. Partial similarity does not imply functional equivalence between the sequences compared. Accounting for full sequence similarity is important, as exemplified by the human La protein (Sjögren's syndrome antigen B) whose homologs in fungi are not essential but which is critical for the survival of mESC cells [17]. The fact that the yeast sequence (275 amino acids long) is much shorter than the human and mouse proteins (408 and 415 amino acids long, respectively), probably accounts for these striking functional differences. A stricter definition of homology requires the identical domain organization of the compared proteins [18], which can be approximated to sequence similarity extending over the full length of the compared sequences. To do this, we required that fewer than 30 amino acids were left unmatched at the C- or N-termini of either of the two compared sequences.

With this additional constraint, differences in the distribution of homologues emerged (Figure 4). Relative to the complete set described above, a smaller proportion of proteins from genes with large expression changes was found to have full-length homologues in non-mammalian species (with the largest difference in the fly). This indicates that the genes with large changes in gene expression are enriched in genes which appeared after the emergence of metazoans (especially of arthropods), and before the mammals' radiation.

For genes with the smallest expression changes, an increased proportion of proteins have full-length homologues in all species. This observation agrees with the hypothesis that the smallest changes in expression would be observed in housekeeping genes, which would be expected to be conserved across a wide range of species.

### 2.4. Selection of a small set of probe sets and genes

To focus on a small set of genes for illustration, comparison to other analyses, and to suggest targets for experimental work, we selected probe sets showing consistent expression changes across replicates in the three cell lines analyzed. We used RMA [19] to normalize the data for the first three time points in each of the three time series (9 microarrays for each cell line, as triplicate arrays were run for each time point). Then for each cell line, we used SAM [20] to identify the top 100 probe sets with the most significant changes in gene expression between 0 h and 6 h, and between 0 h and 12 h, with separate analysis of the MOE430A and MOE430B arrays.

SAM analysis of the MOE430B array indicated much higher false discovery rates than were observed for the MOE430A array (see Methods for details). As the MOE430A array measures genes that are generally better characterized than those on MOE430B, we proceeded to report only the analysis of MOE430A.

**Figure 5:**
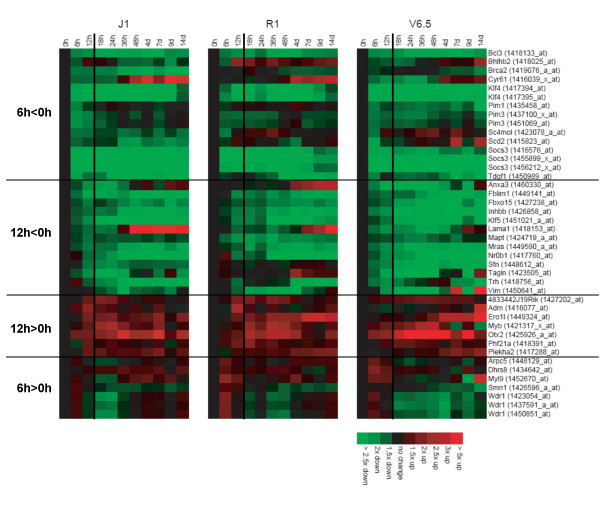
**Signal values of 42 selected MOE430A probe sets in the three time series (R1, J1, and V6.5)**. Gene names and probe set identifiers are indicated on the left. Relative to their signal value at time 0 h, top 15 probe sets displayed a significant signal decrease at 6 h, next 13 displayed a significant signal decrease at 12 h, next 7 displayed a significant signal increase at 12 h, and bottom 7 displayed a significant signal increase at 6 h, in at least two of the three cell lines analyzed.

We compared the top 100 lists for the three cell lines to select probe sets present in at least two out of three lists with fold changes in the same direction for each of the 6 h/0 h and 12 h/0 h analyses. This condition was fulfilled by 15 probe sets corresponding to 11 genes down-regulated at 6 h, for 13 probe sets (13 genes) down-regulated at 12 h, for 7 probe sets (7 genes) up-regulated at 12 h, and for 7 probe sets (5 genes) up-regulated at 6 h (see Figure 5 and Table 1). In total, we selected 42 probe sets corresponding to 36 genes for further analysis. When these probe sets were highlighted on the expression fold change graph (Figure 3), they were found towards the extremes of the distribution, indicating a degree of agreement between the two methods.

To allow statistical analysis of over-represented gene function in genes selected using SAM, we generated a longer list of genes present in the top 1,000 of at least two of the three time series for the 0 h/6 h and 0 h/12 h SAM comparisons, with fold changes in the same direction. This new list was analyzed with GOstat. In the 0 h/6 h comparison, a total of 163 up-regulated probe sets were selected. Over-represented GO terms were "mRNA metabolism" (P-value = 0.0012) and related terms (for example, "nuclear mRNA splicing, via spliceosome", P-value = 0.03), "actin cytoskeleton" (0.02), "nucleic acid binding" (0.03), and "regulation of transcription" (0.05). In the 0 h/6 h comparison, a total of 123 down-regulated probe sets were selected. GO terms over-represented were "sterol metabolism" (0.0004) and related, "immune cell activation" (0.006), and "organ development" (0.00856).

In the 0 h/12 h comparison, a smaller set of statistically significant GO terms was observed. The 244 selected up-regulated probe sets displayed no significant GO term. The 113 down-regulated probe sets had over-represented GO terms related to development, for example "organ morphogenesis" (0.002), "growth factor activity" (0.008), and "angiogenesis" (0.0004).

Functions related to cytoskeleton and transcriptional regulation were highlighted both here and in the broad functional analysis (section 2.2) confirming the relative equivalence of both analyses. The presence of mRNA related functions in the up-regulated probe sets in this analysis is however interesting as suggests that splicing has an important role in the control of mESC differentiation.

### 2.5. Analysis of 36 selected proteins

**Figure 6:**
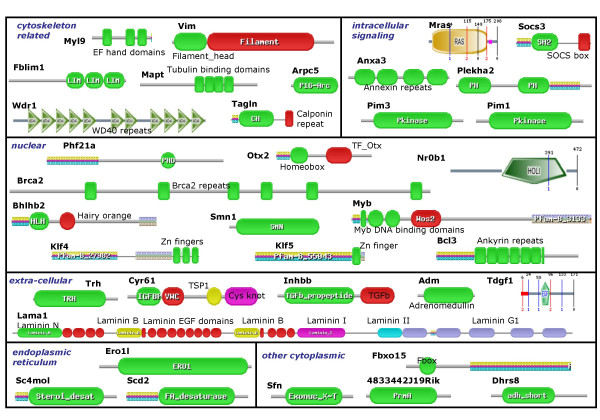
**Domain organization of the 36 selected proteins**. Illustrations are adapted from Pfam [67] or SMART [66]. Names close to or inside the domain pictures correspond to Pfam domain identifiers or, in the case of Tdgf1, Wdr1, Nr0b1, and MRas, to SMART domain identifiers. Three-coloured boxes correspond to Pfam-b domains that are automatically predicted. All sequences are at the same scale except Lama1.

**Figure 7:**
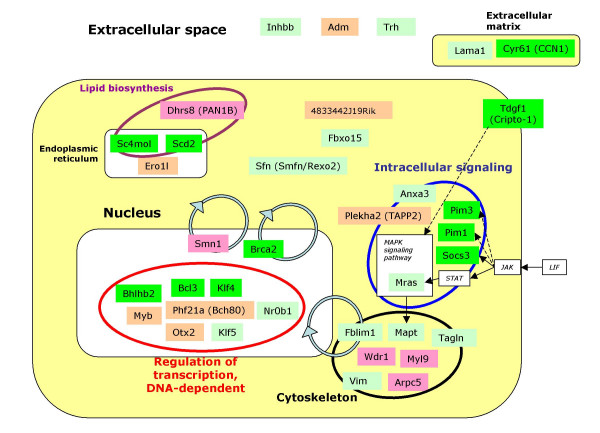
**Function and sub-cellular localization of the 36 selected proteins**. The colours of the labels indicate the behavior of the corresponding probe sets (dark-green: down-regulated at 6 h; light green: down-regulated at 12 h; tan: up-regulated at 12 h; pink: up-regulated at 6 h). Pathways and other related proteins are indicated with white labels. Circular arrows indicate transport between cytoplasm and nucleus. Solid and broken arrows indicate direct activation and activation through intermediate proteins, respectively. Information about the JAK-STAT and MAPK pathways was taken from the references listed in this manuscript or from the KEGG database [73].

The domain organization of the 36 selected gene products are represented in Figure 6. Their functions, subcellular localization, and up- or down-regulation are schematized in Figure 7. The relation of some of these genes and proteins to differentiation is known, but some are poorly characterized.

The phylogenetic analysis of their sequences (following the methodology described in section 2.3; details in Methods section) indicated that sequence similarity is found for most of these proteins across the metazoan species studied (for example, 30 of the 36 proteins, 83%, are similar to fly proteins). However, full-length sequence similarity was much more restricted (only five sequences, 14%, to fly proteins) [see additional file 2]. These proteins contain many domains general to all metazoans, accounting for the high levels of partial sequence similarity (see Figure 6), but their domain organization is more specific. One such example is the Nr0b1 protein: towards the C-terminus it contains a HOLI domain, which is also found in fly and worm proteins, but its N-terminal sequence (amino-acids 1–257), and thus its global domain composition, are unique to mammals. Another example is Plekha2, which has two Pleckstrin Homology (PH) domains (Figure 6); while these domains are present in the eukaryote phylum down to the yeasts, the sequence between the two PH domains (residues 116–198), present in amphibians and fish (*Xenopus laevis *and *Danio rerio*), is missing from fly, worm, and yeast sequences.

This analysis shows that the protein products of *Myl9, Tagln, Sc4mol*, and *Wdr1*, have full-length similar sequences in the yeast, which argues against their involvement in stem cell related functions. Conversely, the protein products of *Adm *and *Trh*, do not appear to have any significantly similar sequence (even partially similar) in the worm and the fly, indicating they probable appeared with the emergence of arthropods. Thus, their role in stem cell (specific) related processes seems more plausible. All 36 proteins have very similar sequences in human, with at least 28 having full-sequence-similarity.

The following sections describe these 36 proteins, their functions, sub-cellular localization, and known or possible relation to mESC differentiation. Relevant protein synonyms are indicated within brackets.

### 2.5.1. Intracellular signaling related proteins

Strikingly, five out of six protein products related to intracellular signaling were associated with probe sets down-regulated at 6 hr (Pim1, Pim3, SOCS3) or later at 12 hr (Anxa3, Mras); only *Plekha2 *(TAPP2) was up-regulated (Figure 6). The response of some of these genes to LIF is known. *SOCS-3*, suppressor of cytokine signaling-3, a negative regulator of the insulin receptor signaling pathway [21], is known to be transcriptionally activated by LIF [22]. This fits with the observed reduction in its expression upon LIF removal. The role of SOCS-3 in differentiation remains unclear, as it has been observed to act both as a promoter [23] and as a repressor of differentiation in mESC [24].

*Pim1*, proviral integration site 1, and *Pim3*, proviral integration site 3, belong to a family of serine/threonine kinases. PIM genes (*Pim1, Pim2, Pim3*) were initially discovered through proviral insertional activation by murine leukemia virus and have been shown to cooperate with c-MYC in murine leukemia. *Pim *genes are related to multiple growth processes including cytokine-mediated cell growth and differentiation of bone marrow cells [25]. However, *Pim *knock-out mice are viable and fertile, which makes it unlikely that these genes play an important role in mESC differentiation. Induction of *Pim *expression is likely mediated by JAK/STAT signaling [26] and that pathway is induced by LIF, so the observed decrease in expression of both *Pim1 *and *Pim3 *is consistent.

*Mras/Rras3*, muscle and microspikes RAS, is a RAS small GTPase. The human *Mras *is associated with the biogenesis of the actin cytoskeleton in muscle cells and its product interacts with the RAS-binding domain of a multitude of proteins including RAF1 and RALGDS [27]. When activated by STAT3 [28], it mediates the activation of MAPK in a cell dependant manner [29], thus linking the JAK/STAT and RAS/MAPK pathways. This agrees with the observed down-regulation of *Mras *as LIF induces JAK/STAT, and STAT3 induces this gene.

*Plekha2 *(TAPP2) is an adaptor protein that can interact, through its two PH domains, with the lipids of the plasma membrane produced by the PI3 kinases, but its role is unknown [30]; here we propose that it is associated with early ESC differentiation.

### 2.5.2. Cytoskeleton related proteins

Seven protein products were related to the cytoskeleton: three associated with probe sets up-regulated at 6 h (*Wdr1, Arpc5*, and *Myl9*), and four associated with down-regulated probe sets (*Fblim1, Vim *(vimentin)*, Tagln *(transgelin), and *Mapt *(microtubule-associated protein tau)). The three down-regulated proteins are found in all eukaryotes and therefore their importance to ESC development is dubious: WDR1 protein (also AIP1, Actin interacting protein 1) has been associated with cell morphologic changes [31], ARPC5 is a component of the Arp2/3 complex, an actin filament nucleator that activates regulated actin assembly in response to extracellular signals in eukaryotic cells [32], and *Myl9 *(myosin, light polypeptide 9, regulatory) is characterized by its similarity to the human *Myl9 *or MLC2 [33], the myosin light chain 2.

The protein encoded by *Fblim1 *is known as Migfilin and acts as mediator between the plasma membrane and the actin cytoskeleton. Migfilin is also involved in cell morphology [34]. The other three down-regulated have developmental roles, but they are markers of differentiated cells: Vimentin of neuroepithelial cells, Tau of retinal ganglion cells, and Transgelin (SM22alpha) of smooth muscle cells.

### 2.5.3. Nuclear proteins

The importance of transcriptional regulation for the control of mESC differentiation is exhibited in the high percentage of nuclear proteins in our selected set. Four proteins corresponded to up-regulated probe sets: *Smn1, Phf21a, Myb*, and *Otx2*. Another six proteins corresponded to down-regulated probe sets: *Brca2, Bhlbh2, Bcl3, Klf4, Klf5*, and *Nr0b1*.

Eight of these ten proteins regulate transcription through DNA interaction. For example, the transcription factor Klf4 (down-regulated at 6 hr), Kruppel-like factor 4, inhibits murine embryonic stem cell differentiation [23], which agrees with the observed down-regulation. It has been described as inducing and repressing a number of signaling pathways that control macrophage activation [35]. *Otx2 *encodes a homeobox domain containing protein, which is a regulator of neurogenesis in the developing brain in mouse embryo [36].

The two that are not transcription factors, SMN1 and BRCA2, can both shuttle between nucleus and cytoplasm. SMN1 is present in cytoplasm, nucleoplasm, and in the Cajal bodies where it is needed for the biogenesis of spliceosomal small nuclear ribonucleoproteins [37]; knock-out of these gene leads to massive cell death in early mice embryos [38]. BRCA2 is involved in DNA repair and can be transported to the cytoplasm probably as a way to control its function [39].

### 2.5.4. Extracellular proteins

Six extracellular proteins were in our list of selected proteins, all but one down-regulated. Three of them are related to a receptor binding function: *Trh *(tyrothropin releasing hormone)*, Lama1 *(laminin alpha-1), and *Tdgf1 *(Cripto). The latter is an indirect activator of the Ras/MAPK pathway [40] and well known as involved in the determination of the anterior-posterior axis in the mouse embryo [41]. Laminin alpha-1, is also involved in embryonic patterning and is one of the few essential extracellular matrix proteins in early embryogenesis [42].

The others are *Inhbb *(inhibin beta-B) that acts as activin-B, a homodimer of two Inhbb, and two angiogenic factors,* Adm *(adrenomedullin) [43], and *Cyr61 *(CCN1), the latter being present in the extracellular matrix [44].

### 2.5.5. Other intracellular proteins

There were eight other intracellular proteins in our list: four cytoplasmic, *Fbxo15, 4833442J19Rik, Sfn *(Smfn/Rexo2), and *Dhrs8*, and three located in the ER *Ero1l*, *Sc4mol*, and *Scd2*. Two of the latter, *Sc4mol *(ERG25) and *Scd2*, were up-regulated at 6 h and are related to lipid biosynthesis, whereas up-regulated *Ero1l *codes for an oxidoreductase involved in oxidative ER protein folding [45]. ERG25 is a sterol C-4 methyloxidase involved in the biosynthesis of sterol [46]. SCD2 is a stearoyl-CoA desaturase involved in the biosynthesis of monounsaturated fatty acids [47]. Lipids, besides their function in modulating membrane function, can also act as second messengers in developmental signaling. Changes in the expression of genes coding for enzymes involved in their biosynthesis could reflect alterations in the cellular membrane related to the ESC differentiation process.

Cytoplasmic *Sfn *(Smfn/Rexo2) is a 3' to 5' exonuclease of small (< 5nt) poly-nucleotides, hypothesized to be involved in nucleotide recycling, and conserved in yeasts and in *Escherichia coli *[48]. Fbxo15 (Fbx15), Fbox protein 15, is a protein of unknown function, known to be target of Oct3/4 but not essential for development [49]; as it contains an F-box domain this protein could recruit a target protein for ubiquitination. Dhrs8 (PAN1B) is an enzyme with 17β-hydroxysteroid dehydrogenase activity also involved in lipid biosynthesis [50]; 17β-dehydrogenases play roles in the activation and inactivation of both estrogens and androgens.

In summary, our methodology primarily selected proteins involved in intracellular signaling, cytoskeletal control, and regulation of transcription. None of the 36 proteins appear to have functions related to translation and amino-acid biosynthesis, few are secreted, and the few enzymes selected are involved in lipid biosynthesis.

## 3. Discussion

Mouse and human ESCs have been the subject of gene expression profiling in previous studies using DNA microarrays, Serial Analysis of Gene Expression (SAGE), and Expressed Sequence Tag (EST) library generation (see *e.g. *[7] and references therein). However, most of those studies compared undifferentiated ESCs to their fully differentiated derivatives, and none followed gene expression dynamics during the first 12 hours of the differentiation process. Bhattacharya et al. [51] compared five lines of hESCs to 8-day old EBs. Dvash et al. [52] compared H9 hESCs to 2 day, 10 day, and 30 day old EBs. Palmqvist et al. [7] compared R1 mESC with ESC differentiated for 18 hours and 72 hours. Sekkai et al. [6] compared undifferentiated Gs2 hESC with ESC differentiated for 16, 24, and 48 hours. The objective in most of these works was to detect genes turned off or on temporarily during the early differentiation period. Here, we have carried this idea further by obtaining measurements of mESC differentiating into EBs during a two-week period, including measurements of gene expression as early as 6 hours and 12 hours after differentiation was started.

Our analysis examined three genetically distinct mESC lines (R1, J1, and V6.5) with all measurements in biological triplicate, both to assess the robustness of the observed changes and to avoid biases due to particular mouse strains. We showed the existence and duration of an early period in the time series of differentiation prior to the onset of differentiation. This is important for the characterization of gene expression in ESCs undergoing differentiation before they differentiate into another cell type, or a mixture of cell types. Clustering the data both by expression levels of transcription factor genes (Figure 1) and expression patterns of mESC markers (Figure 2) suggested that the first three time points in the time series (*i.e*. the start point, 6 hours, and 12 hours) corresponded to undifferentiated stem cells in which major expression changes had not yet begun. We took this as an indication that the set of genes with expression changes during that early period would include genes that trigger the differentiation process.

Next we studied patterns of gene expression during this early period compared to the initial values in the undifferentiated state. We used the changes with respect to the signal at initial time point of each series (Figure 3). We observed a distinct functional profile and phylogenetic distribution for the genes with large expression changes during the period under study, which were selected as possibly involved in the differentiation process.

Although this analysis provided a way to test several types of gene expression profiles in a simple manner, we chose a more sensitive method of gene expression analysis to select a list of exemplar genes, SAM [20], which allows the detection of consistent changes in gene expression using the variation between replicates.

Using SAM analyses of gene expression to contrast the initial condition with either the 6 h or the 12 h time point, we focused on 42 probe sets (representing 36 protein products) with the most consistent changes of gene expression in at least two of the three cell lines analyzed (Figure 5). These probe sets had functional and phylogenetic properties consistent to those of the genes selected as expressed and whose expression changed the most in the broad analysis.

Among the genes selected, we note a majority of functions related to the control of transcription (*Klf4, Klf5, Bcl3, Myb, Otx2, Bhlhb2, Phf21a, Nr0b1*), intracellular signaling (*Socs3*, *Anxa3, Mras, Pim1, Pim3*), and cytoskeletal related (*Fblim1, Arpc5, Tagln, Wdr1, Myl9, Vim, Mapt*) (Figure 6).

Gene expression changes must in large part be modulated through transcription factors, thus we would expect such functions to be well represented in the lists of changing genes. We note that this is one of the most represented functions and that we found both up-regulated and down-regulated transcription factors. This suggests that, at least in the 0 h–12 h frame, the transcriptional control is not limited to a small number of transcription factors.

Of the 36 protein products studied, six were associated with the related JAK-STAT and MAPK signaling pathways (*Tdgf1, Socs3, Pim1, Pim3, Mras, Mapt*), which are activated by LIF. These six genes were down-regulated. As these pathways transduce a large variety of external signals, this down-regulation must have a major effect on the cell state.

The abundance of cytoskeletal proteins is not surprising in light of the number of intracellular signaling proteins detected. It is well known that cytoskeletal proteins, besides their structural function, serve to localize intracellular signal pathways (two such examples in ESC are Paxillin [53] and beta 1 integrin [54]).

The broad reduction in the expression levels of genes related to the extracellular matrix (two of which, *Lama1 *and *Cyr61*, are among the genes most significantly down-regulated) reflects the changes in the cell interaction properties that happen upon differentiation of mESC. ES cells are cultured as an attached monolayer, but their differentiated derivatives grow as unattached spheres. Some studies are starting to shed light on how the interrelation between mESC and their environment, mediated through proteins of the extracellular matrix, controls their self-renewal [55]. Our study suggests *Lama1 *and *Cyr61 *as candidate genes for the study of this process.

The absence of certain functions from the genes selected in our broad and focused analyses also provides information about the timing of cell processes involved in ESC differentiation. Modifications in the mobility of chromatin interacting proteins are known to occur during loss of pluripotentiality of ESC [56]. However, we did not observe expression changes in the genes for chromatin interacting proteins other than BRCA2, which is implicated in chromatin remodeling [57]. Neither did we observe large modifications in the expression of genes coding for surface receptors; such changes are occurring later in human ESC differentiation into embryoid bodies [52]. Apparently, the only proteins with enzymatic activity in our sort list of 36 were two endoplasmic reticulum resident proteins, *Sc4mol *(ERG25) and *Scd2*, both up-regulated at 6 hours and related to lipid biosynthesis. This may indicate a lipid involvement in signaling associated with the differentiation process.

We hypothesized that the genes that control ES differentiation are likely to have arisen in organisms which have stem cells, and thus to be found only in metazoans. Other genes which control specific mammalian aspects of ES differentiation may show a distribution specific to mammals. To examine possible correlation between genes with changing expression and the phylogenetic distribution of their protein products, we searched for their protein homologues in model organisms, distinguishing between homologues with total or partial sequence similarity. The contrast between the conservation in arthropods and other eukaryotic organisms of the genes of changing expression (Figure 4) suggests that our data and approach are useful for detecting genes that might be associated with stem cell functions.

A similar analysis for the 36 mouse protein products with the most significant changes [see additional file 2] indicates that several of the proteins have no full-length similar sequences in the yeast. Others that have more general functions (e.g. DNA binding, protein phosphorylation) are not exclusive to mammals as they contain domains that are found in lower eukaryotes such as yeast (*e.g*. PH, RAS, LIM; Figure 5). Their full sequence domain organization is more revealing of their taxonomic specificity: while 31 of these 36 mouse genes had high similarity to fly sequences, only 5 of those had full-sequence similarity.

Since the set of 36 mouse gene products studied is highly replicated in the human genome (with highly similar sequences for all 36 and full-length similarity for 28), we suggest that these 36 genes will include genes relevant both for mouse and human ESC differentiation. However, the generalization of results obtained in mESCs to conclusions about hESCs must be approached with caution as there are many obvious differences between human and murine ESCs, starting with the inability of maintaining hESC pluripotentiality with LIF.

**Figure 8:**
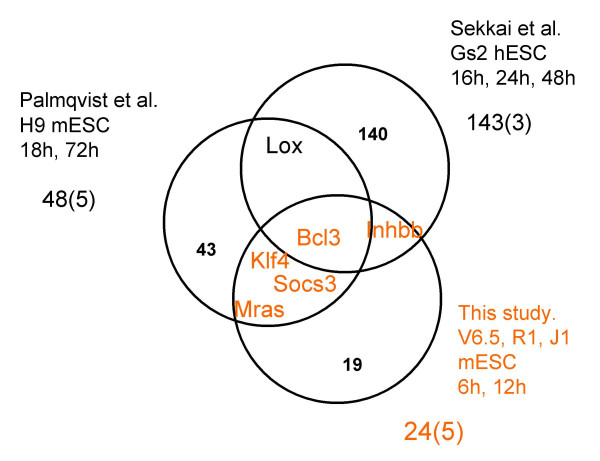
**Correspondence between down-regulated genes from this study with two previous studies of mESC differentiation**. Venn diagram showing the genes found in at least two studies. We note that the cell lines, conditions, and gene selection procedures for these three studies are very different. Palmqvist *et al*.: 48 genes from Table 3 in [7]. Sekkai *et al*.: 143 genes from Table S1 of [6]. This study: 24 genes down-regulated in at least two cell lines at 6 hr or 12 hr.

In summary, our study provides for the first time a functional and phylogenetic signature associated with gene expression patterns in early differentiating mESC. This is not surprising since this study differs from previous studies in cell types and time frame analyzed. To contrast our results to those from previous studies, we compared our list of 24 down-regulated genes at 6 and 12 hours from MOE430A (Table 1) with the list of 143 down-regulated genes at 16, 24, and 48 hours from Sekkai et al (Gs2 hESC; Supplementary Table 1 from [6]) and the list of 48 down-regulated genes at 18 and 72 hours from Palmqvist et al (H9 mESC; Table 3 in [7]) (Figure 8). Only one gene, *Bcl3*, was identified by all three analyses, although the importance of this gene for embryo development is doubtful since knock-out mice for this gene are viable only showing immunological defects [58]. Our list has five genes in common with the other two studies, more than they share in common with each other. However, differences in experimental design and analyses complicate the comparison to previous studies, especially since we focused on an earlier time frame.

In this respect, we note that three cytoskeletal genes that we found to be up-regulated at 6 hr (*Wdr1, Myl9, Arpc5*) are down-regulated in our data at 18 hr and 24 hr (Figure 4). Studies not including the early time points would miss the initial period of up-regulation, and report these genes as decreasing with differentiation. Similarly, *Bhlhb2 *and *Sc4mol *are down-regulated at 6 hr and later up-regulated (Figure 5). We take this as an indication that our study contributes useful information not provided by earlier analyses.

Differences observed between the results obtained from each cell line provide justification for our approach of using multiple cell lines. For example, in our SAM analysis (section 2.4) two probe sets indicated significant gene expression changes during the first 6 hours for the *Hnrpdl *gene (heterogeneous nuclear ribonucleoprotein D-like, coding for protein JKTBP, an mRNA interacting protein that shuttles between nucleus and cytoplasm [59]). However, the gene was up-regulated in R1 and down-regulated in V6.5. An analysis using either one or the other cell type would report opposite results. By using the consensus of three cell lines we expect to have increased the relevancy of our observations.

## 4. Conclusion

In this work, we have proposed a set of functions and genes whose protein products might participate in processes relevant to the early differentiation of mammalian ESCs. These genes probably participate in resetting the cellular mRNA set, while intercellular interactions and protein production are temporarily slowed down before changes in chromatin remodeling and surface receptors occur.

We have focused on the early stages of mESC differentiation. Further work is possible to study later stages, apply other methodologies to study gene expression, and to compare to other microarray experiments measuring stem cell gene expression changes with differentiation. To facilitate these future studies we have made our DNA microarray data available both as raw data and in the context of a database of stem cell gene expression data [1]. We expect that this resource will constitute an advance in the field of stem cell research.

## 5. Methods

### 5.1. mESC culture

Three mouse ES cell lines derived from different mouse strains were used. J1 is from 129Sv/J, R1 is from a cross between 129X1 and 129S1, and V6.5 is from a cross between 129Sv/J and C57BL/6. By using several cell lines we will better be able to identify the genes critical for ES differentiation as these should be common for all three.

R1, J1, and V6.5 mESCs were maintained at 37°C humidified air with 5% CO2 on a layer of irradiated DR4 mouse embryo fibroblasts (MEFs) and fed daily with a complete change of ESC maintenance medium consisting of high-glucose Dulbecco's modified Eagle's medium (DMEM) (all reagents obtained from Invitrogen Life Technologies, Burlington, Ontario, Canada, unless otherwise indicated) supplemented with 15% ESC Characterized fetal bovine serum (FBS; Hyclone, Logan, Utah, USA), 0.1 mM nonessential amino acids, 2 mM glutamine, 1,000 U/ml LIF (Chemicon, Temecula, California, USA), 100 U/ml penicillin, 100 μg/ml streptomycin, and 0.1 mM 2-mercaptoethanol (Sigma, Oakville, Ontario, Canada). mESCs for gene expression profiling were from passage 13 and had been frozen at 10^6 ^cells per vial. Cells were passaged every second day to maintain undifferentiated state. To passage cells, a single-cell suspension was generated by treatment with 0.25% trypsin and 1 mM EDTA (T/E) for 5 minutes until cells detached from the culture vessel surface. Trypsin activity was then quenched with an equal volume of mESC media. The cells were centrifuged at 1,100 rpm for 4 minutes and resuspended in ESC media. Viable cells were plated at 1 × 10^6 ^per 100-mm dish on irradiated MEFs and cultured for a further 48 hours at 37°C, 5% CO_2 _before RNA isolation by RNeasy Kit (Qiagen, Mississauga, Ontario, Canada). In subsequent experiments, all cells used were within five passages of initial thawing. DR4 MEFs were maintained at 37°C humidified air with 5% CO_2 _in DMEM supplemented with 10% FBS, 100 U/ml penicillin, 100 μg/ml streptomycin, 0.1 mM nonessential amino acids, and 2 mM glutamine. Cells to be irradiated were trypsinized, resuspended in 2 ml of medium, and exposed to 60 Gy from an X-ray source before replating for use as feeder cells.

### 5.2. Preparation of mESCs for differentiation

mESCs were thawed and cultured for two passages over 96 hours as described above. To prepare cells for differentiation, they were harvested and washed as described above, resuspended in ESC maintenance medium, and preplated on tissue culture plates for 1 hour at 37°C, 5% CO_2 _to deplete contaminating DR4 MEFs. Cells were pelleted by centrifugation as above, and viable cell numbers were determined. The preplated mESCs were passaged at a density of 1 to 2 × 10^7 ^cells per low adherence p100 tissue culture dish in the presence of MEF media. Fifty percent media exchanges were performed every second day. Cells were harvested at the indicated timepoints and RNA isolated as above.

### 5.3. DNA microarray experiments

We extracted mRNA from three mESC cell lines (R1, J1, V6.5) at 11 time points following removal of LIF from a feeder-cell-free culture. cDNA generated from the mRNA was hybridized to the Affymetrix MOE430 GeneChip set in biological triplicate.

The data for each of the three time series are deposited in the StemBase database of stem cell gene expression [1,60] under the experiment identifiers E165, E201, and E113, for cell lines R1, J1 and V6.5, respectively. The data are also deposited in the GEO repository of gene expression data [61] with the series accession identifiers GSE2972, GSE3749, and GSE3231, respectively.

#### 5.4. Computational analyses

Expression data, obtained in biological triplicate, were processed as follows. For the broad analysis of gene function changes (Figure 3) [see Additional file 1] the MAS5 signal data replicates were averaged, then data from MOE430A and MOE430B GeneChips was pooled and scaled per sample by normalizing to the trimmed mean for all probe sets in the sample (98%). MAS5 calls were collated by majority vote, that is, a probe set was considered to have a P call (Present) if MAS5 generated a P call in at least two of the three replicates.

For other fold change analyses, we normalized the Affymetrix CEL files using RMA [19] implemented in Bioconductor [62]. For the study of the complete time series (Figures 1, 2, and 5), the data from all 11 time points were normalized for each cell line. For the identification of coherent gene expression changes during the first 12 hours of the series (sections 2.4 and 2.5) the data from the first three time points were normalized for each cell line. We used the implementation of the SAM algorithm [20] from the Bioconductor siggenes package to identify probe sets whose signal showed significant change between 0 h and 6 h, and between 0 h and 12 h, in each of the three cell lines. We selected probes sets in the top 100 most significantly changed list for at least two of the three cell lines. The analysis was done separately for the MOE430A and MOE430B GeneChip data. SAM reported higher false discovery rates (FDRs) for the lists from the MOE430B array than for the MOE430A lists. For example, for the analysis of V6.5 between 0 hours and 6 hours, the false discovery rate reported for the top ~1000 significantly changed probe sets is of 22% for MOE430A and of 53% for MOE430B. Accordingly, the level of agreement between cell lines was also much lower in the MOE430B than in the MOE430A, and therefore we concentrated on the results from the MOE430A GeneChip. FDRs for the MOE430A top 100 lists was in the 9–16% range.

The gene name, gene symbol, and GO terms corresponding to each probe set were retrieved from the NetAffx annotation database (April 2005 version; [63,13]). Additional GO terms were obtained by examination of related databases assisted by automated data mining procedures [64]. We retrieved amino acid sequences and gene information from NCBI [65]. The protein domain architecture was predicted using domain databases (SMART [66], Pfam [67], and InterPro [68]).

GOstat [11,69] was used to examine groups of probe sets (both in the broad and in the focused analyses) for over- and under-represented GO terms, using MGI [70] as the GO to gene association database, and using the Benjamini false discovery rate correction. In the broad functional analysis (section 2.2), probe sets were provided as gene identifiers, and the full list of 16,752 probe sets selected as expressed in at least one condition was provided as a reference set. Using this alternative reference set prevents bias arising from the fact that many classes of genes (for example, olfactory receptors) are not expressed in mESCs. Use of the whole array gene set as a reference would have resulted in such GO terms being highlighted as significantly under-represented. In the focused analysis (section 2.4), gene names were provided as gene identifiers, and the reference set was the full mouse genome. We reported GO terms selected among the best 30 of each analysis and involving a minimum of six genes.

We used BLAST [71] to perform homology searches against the SPtrEMBL protein database [14] (section 2.3) and against the NCBI's organism databases [61] (section 2.5) [see additional file 2], to study the phylogenetic distribution of the genes across model eukaryotic organisms.

## 6. Abbreviations

EB, Embryoid Body

ESC, Embryonic Stem Cell

EST, Expressed Sequence Tag

GO, Gene Ontology

hESC, Human Embryonic Stem Cell

LIF, Leukemia Inhibitory Factor

MEF, Mouse Embryo Fibroblasts

mESC, Mouse Embryonic Stem Cell

SAGE, Serial Analysis of Gene Expression

## 7. Authors' contributions

KHS, CJP, GP, CP, EMM, and MAA participated in the computational analysis. MAR designed the experimental data collection. PAC managed the experimental stem cell data collection. MAA designed the computational study and drafted the manuscript. All authors read, corrected, and approved the final manuscript.

## Supplementary Material

Additional file 1 Normalized signal values. Normalized signal values at points 0 h, 6 h and 12 h, in the three time series, for 16,752 probe sets. This table is also provided at [74] both in XLS (Excel spreadsheet) and in tab delimited text formats. Click here for file

Additional file 2 Sequence similarity analysis of the 36 protein products. Table columns are: **Protein **name; **gi **identifier of the protein in Genbank; **length **of the protein. Then, for each of a series of organisms or taxonomic divisions (*Homo sapiens, Rattus norvegicus, Xenopus laevis, Danio rerio, Drosophila melanogaster, Caenorhabditis elegans*, *Saccharomyces cerevisiae, Bacteria, Archaea*) the following information of a match of sequence similarity: **subject_id**, the Genbank identifier of the match; **length **of the match; **e_val**, the E-value of the match; **alignment**, for numbers with the start and end amino acid positions of the alignment between query and subject, respectively. Cell background colour codes the match type with orange for full length high sequence similarity (E-value < = 1e-6; less than 30 amino acids left out of the alignment and the N- and C-termini of subject and query), yellow for high sequence similarity (E-value < = 1e-6), or white for low or no sequence similarity (E-value > 1e-6). This table is also provided at [74]. Click here for file

## Figures and Tables

**Table 1:** Probe sets selected by SAM analysis (section 2.4).

**key**	**Probe set id**	**Gen**	**Gen description**	**Function/process/component (GO)**^1^
D6	1418133_at	Bcl3	B-cell leukemia/lymphoma 3	DNA binding; transcription factor activity; regulation of transcription, DNA-dependent; *nucleus;*
D6	1418025_at	Bhlhb2	basic helix-loop-helix domain containing, class B2	DNA binding; protein binding; nucleus; regulation of transcription, DNA-dependent; transcriptional repressor activity; negative regulation of transcription, DNA-dependent;
D6	1419076_a_at	Brca2	breast cancer 2	nucleic acid binding; protein binding; nucleus; cytoplasm; DNA repair; response to DNA damage stimulus; *double-strand break repair via homologous recombination; synaptonemal complex; single-stranded DNA binding; DNA replication; chromatin remodeling; apoptosis; regulation of S phase of mitotic cell cycle; mitotic checkpoint; transcriptional activator activity; regulation of transcription;*
D6	1416039_x_at	Cyr61	cysteine rich protein 61	regulation of cell growth; patterning of blood vessels; protein binding; insulin-like growth factor binding; extracellular; extracellular space; chemotaxis; cell adhesion; heparin binding; growth factor binding; *sensory perception;*
D6	1417394_at	Klf4	Kruppel-like factor 4 (gut)	nucleic acid binding; DNA binding; nucleus; regulation of transcription, DNA-dependent; zinc ion binding; *transcription factor activity; transcriptional activator activity; transcriptional repressor activity; negative regulation of transcription, DNA-dependent;*
D6	1417395_at			
D6	1435458_at	Pim1	proviral integration site 1	protein kinase activity; protein serine/threonine kinase activity; protein-tyrosine kinase activity; ATP binding; nucleus; protein amino acid phosphorylation; cell growth and/or maintenance; kinase activity; transferase activity;
D6	1437100_x_at	Pim3	proviral integration site 3	protein kinase activity; protein serine/threonine kinase activity; ATP binding; protein amino acid phosphorylation; kinase activity; transferase activity;
D6	1451069_at			
D6	1423078_a_at	Sc4mol	sterol-C4-methyl oxidase-like	C-4 methyl sterol oxidase activity; catalytic activity; endoplasmic reticulum; plasma membrane; fatty acid metabolism; metabolism; steroid metabolism; integral to membrane; sterol biosynthesis; oxidoreductase activity;
D6	1415823_at	Scd2	stearoyl-Coenzyme A desaturase 2	fatty acid biosynthesis; superoxide metabolism; lipid biosynthesis; stearoyl-CoA 9-desaturase activity; copper, zinc superoxide dismutase activity; iron ion binding; oxidoreductase activity; metal ion binding; oxidoreductase activity, acting on paired donors, with oxidation of a pair of donors resulting in the reduction of molecular oxygen to two molecules of water; endoplasmic reticulum; membrane; integral to membrane; endoplasmic reticulum
D6	1416576_at	Socs3	suppressor of cytokine signaling 3	regulation of cell growth; signal transduction; intracellular signaling cascade; *regulation of protein amino acid phosphorylation; protein binding; negative regulation of insulin receptor signaling pathway;*
D6	1455899_x_at			
D6	1456212_x_at			
D6	1450989_at	Tdgf1	teratocarcinoma-derived growth factor	activation of MAPK; extracellular; extracellular space; epidermal growth factor receptor signaling pathway; growth factor activity; positive regulation of cell proliferation; determination of anterior/posterior axis, embryo; extrinsic to plasma membrane;
D12	1460330_at	Anxa3	annexin A3	phospholipase inhibitor activity; calcium ion binding; calcium-dependent phospholipid binding; phospholipase A2 inhibitor activity;
D12	1449141_at	Fblim1	filamin binding LIM protein 1	*protein binding; cytoskeleton; cell adhesion; zinc ion binding; regulation of cell shape;*
D12	1427238_at	Fbxo15	F-box protein 15	protein binding; ubiquitin cycle; SCF ubiquitin ligase complex;
D12	1426858_at	Inhbb	inhibin beta-B	hormone activity; extracellular; growth factor activity; cell growth and/or maintenance; growth; *ovarian follicle development; defense/immunity protein activity; cytokine activity; response to external stimulus; protein homodimerization activity; host cell surface receptor binding; positive regulation of follicle-stimulating hormone secretion; negative regulation of follicle-stimulating hormone secretion; negative regulation of hepatocyte growth factor biosynthesis;*
D12	1451021_a_at	Klf5	Kruppel-like factor 5	nucleic acid binding; DNA binding; nucleus; regulation of transcription, DNA-dependent; zinc ion binding; *angiogenesis; transcription factor activity; protein binding; microvillus biogenesis; positive regulation of transcription;*
D12	1418153_at	Lama1	laminin, alpha 1	morphogenesis of an epithelial sheet; receptor binding; structural molecule activity; extracellular matrix structural constituent; protein binding; extracellular matrix; basement membrane; basal lamina; laminin-1; cell adhesion; cell surface receptor linked signal transduction; histogenesis; regulation of cell adhesion; regulation of cell migration; regulation of embryonic development; *establishment of epithelial cell polarity;*
D12	1424719_a_at	Mapt	microtubule-associated protein tau	protein binding; cytoskeletal regulatory protein binding; cytoskeleton; microtubule associated complex; microtubule-based process; microtubule stabilization; *microtubule; actin filament organization; microtubule-based movement;*
D12	1449590_a_at	Mras	muscle and microspikes RAS	GTP binding; small GTPase mediated signal transduction; *small monomeric GTPase activity; RAS small monomeric GTPase activity;*
D12	1417760_at	Nr0b1	nuclear receptor subfamily 0, group B, member 1	DNA binding; transcription factor activity; steroid hormone receptor activity; receptor activity; ligand-dependent nuclear receptor activity; nucleus; cytoplasm; regulation of transcription, DNA-dependent; negative regulation of transcription; transcriptional repressor activity;
D12	1448612_at	Sfn	stratifin	regulation of progression through cell cycle; regulation of cyclin dependent protein kinase activity; protein domain specific binding; cytoplasm;
D12	1423505_at	Tagln	transgelin	muscle development; *NOT cytoskeleton organization and biogenesis*;
D12	1418756_at	Trh	thyrotropin releasing hormone	hormone activity; neuropeptide hormone activity; protein binding; extracellular; extracellular space; thyrotropin-releasing hormone activity; hormone mediated signaling;
D12	1450641_at	Vim	vimentin	structural molecule activity; intermediate filament; type III intermediate filament; intermediate filament-based process; *oxygen transporter activity; oxygen transport;*
U12	1427202_at	4833442J19Rik	RIKEN cDNA 4833442J19 gene	S-adenosylmethionine-dependent methyltransferase activity;nucleus;
U12	1416077_at	Adm	adrenomedullin	hormone activity; neuropeptide hormone activity; extracellular; extracellular space; neuropeptide signaling pathway;
U12	1449324_at	Ero1l/LOC434220	ERO1-like (S. cerevisiae)	endoplasmic reticulum; electron transport; protein folding; transport; membrane; oxidoreductase activity; integral to endoplasmic reticulum membrane;
U12	1421317_x_at	Myb	myeloblastosis oncogene	regulation of cell cycle; G1/S transition of mitotic cell cycle; DNA binding; transcription factor activity; nucleus; regulation of transcription, DNA-dependent; calcium ion transport; cell growth and/or maintenance; viral assembly;
U12	1425926_a_at	Otx2	orthodenticle homolog 2 (Drosophila)	DNA binding; transcription factor activity; protein binding; nucleus; regulation of transcription, DNA-dependent; regulation of transcription from Pol II promoter; development; central nervous system development; organogenesis; anterior/posterior pattern formation; dorsal/ventral pattern formation; transcription regulator activity; forebrain development; midbrain development; eye photoreceptor cell fate commitment; cell fate commitment;
U12	1418391_at	Phf21a	PHD finger protein 21A	transcription; regulation of transcription, DNA-dependent; DNA binding; protein binding; zinc ion binding
U12	1417288_at	Plekha2	pleckstrin homology domain-containing, family A (phosphoinositide binding specific) member 2	phosphatidylinositol binding; nucleus; lipid binding; membrane;
U6	1448129_at	Arpc5	actin related protein 2/3 complex, subunit 5	structural constituent of cytoskeleton; cytoplasm; Arp2/3 protein complex; cell motility; actin filament organization; actin cytoskeleton; DNA binding; electron transport; heme binding; lamellipodium; regulation of actin filament polymerization; regulation of transcription;
U6	1434642_at	Dhrs8	dehydrogenase/reductase (SDR family) member 8	extracellular space; metabolism; oxidoreductase activity;
U6	1452670_at	Myl9	myosin, light polypeptide 9, regulatory	calcium ion binding; muscle myosin; regulation of muscle contraction; muscle development; structural constituent of muscle; myosin; motor activity;
U6	1426596_a_at	Smn1	survival motor neuron 1	nucleic acid binding; RNA binding; nucleus; cytoplasm; mRNA processing; spliceosome assembly;
U6	1423054_at	Wdr1	WD repeat domain 1	actin binding; cytoskeleton; perception of sound; actin cytoskeleton;
U6	1437591_a_at			
U6	1450851_at			

Column 1 indicates probe set behaviour: D6, down-regulated at 6 h; D12, down-regulated at 12 h; U12, up-regulated at 12 h; U6, up-regulated at 6 h. Associated information was taken from NetAffx unless otherwise indicated.

^1^GO-terms in italics were manually derived from database entries linked from NetAffx using the assistance of a data mining method [64]
